# Lysosome function in glomerular health and disease

**DOI:** 10.1007/s00441-020-03375-7

**Published:** 2021-01-12

**Authors:** Catherine Meyer-Schwesinger

**Affiliations:** Institute for Cellular and Integrative Physiology, University Medical Center Hamburg Eppendorf, Hamburg, Germany

**Keywords:** Lysosome, Lysosomal storage disorder, Podocyte, Mesangial cell, Glomerulus, Glomerular endothelial cell, Membranous nephropathy, Focal segmental glomerulosclerosis, Crescentic glomerulonephritis, Diabetic nephropathy

## Abstract

The lysosome represents an important regulatory platform within numerous vesicle trafficking pathways including the endocytic, phagocytic, and autophagic pathways. Its ability to fuse with endosomes, phagosomes, and autophagosomes enables the lysosome to break down a wide range of both endogenous and exogenous cargo, including macromolecules, certain pathogens, and old or damaged organelles. Due to its center position in an intricate network of trafficking events, the lysosome has emerged as a central signaling node for sensing and orchestrating the cells metabolism and immune response, for inter-organelle and inter-cellular signaling and in membrane repair. This review highlights the current knowledge of general lysosome function and discusses these findings in their implication for renal glomerular cell types in health and disease including the involvement of glomerular cells in lysosomal storage diseases and the role of lysosomes in nongenetic glomerular injuries.

## Introduction

The term “lysosome,” Greek for “lytic body” first appeared in print 65 years ago, when De Duve et al. set forward to unravel the intracellular distribution patterns of enzymes in rat-liver by tissue fractionation studies (De Duve et al. 1955). The typical lysosome is a single membrane-bound round structure of about 0.2–0.3 µm in diameter. Each mammalian cell comprises between 50 and 1000 lysosomes distributed throughout the cytoplasm. Lysosomes represent a highly dynamic cellular vesicular compartment within the late endocytic pathway, in which biological macromolecules are degraded (Bainton 1981; de Duve 2005). The cargo degraded by lysosomes differs depending on the route of delivery. The endocytic pathway brings small intracellular molecules from the endoplasmic reticulum (ER) and endocytosed membrane proteins to lysosomes (Pryor and Luzio 2009), the autophagic pathway targets intracellular aggregates and dysfunctional organelles to lysosomes (Jegga et al. 2011), and the phagocytic pathway routes phagocytosed large (≥ 0.5 µm) particles such as bacteria to lysosomes for degradation (Herb et al. 2020). Besides the canonical role of lysosomes for the degradation of waste, emerging studies show a significant contribution of lysosomes to the sensing of the nutrient status of cells, to metabolism, membrane repair, and immune signaling (Saftig and Puertollano 2020). This review will broadly cover general lysosome biology, thereafter, focusing on our existing knowledge about lysosome function in glomerular health and disease. This review is based on many excellent expert reviews on lysosomal biology, we apologize to those many researchers, whose work could not be cited in this context.

## Lysosomal composition and biogenesis

**Fig. 1:**
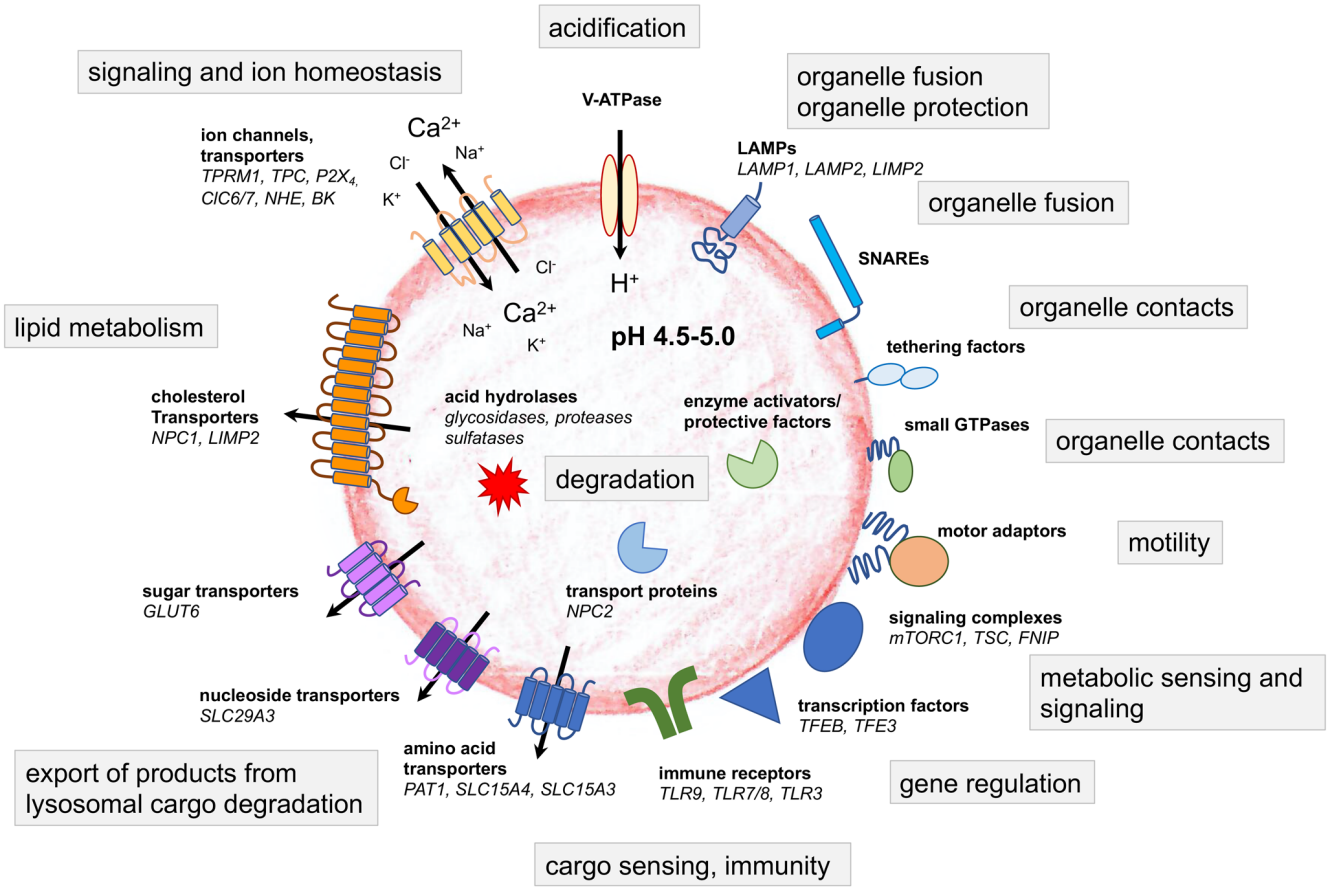
The plethora of lysosomal proteins highlights the complexity of lysosomes and their central role for cellular function, signaling, and metabolism. Modified from Ballabio and Bonifacino 2020

The complexity of lysosomes is emphasized by data from proteome analyses: Roughly 200 glycosylated and nonglycosylated integral membrane proteins have been identified (Fig. 1), which are crucial for the transport of metabolites and for the prevention of lysosomal membrane degradation (Saftig and Klumperman 2009; Saftig et al. 2010). Ion channels and transporters regulate the luminal ion composition (Calcraft et al. 2009; Zhang et al. 2009), tethering factors, and SNARE proteins at the membrane control fission and fusion with communicating cellular compartments (Brocker et al. 2010; Hong and Lev 2014). Lysosomal function is orchestrated by lysosomal degradative enzymes and by lysosomal membrane proteins (Saftig and Klumperma 2009; Schwake et al. 2013). The highly glycosylated transmembrane proteins lysosome-associated membrane proteins 1 and 2 (LAMP1 and LAMP2) are the most abundant lysosomal proteins and are thought to protect the membrane from degradation by lysosomal enzymes (Saftig and Klumperman 2009). The crucial and characteristic acidity of lysosomes is achieved by a dedicated H^+^-ATPase (Graves et al. 2008; Mindell 2012), which maintains the luminal acidic pH to stabilize and mediate the activity of the over 70 lysosomal degradative enzymes that have pH optima ranging from pH 3.5 to pH 5.5 (Lubke et al. 2009). Lysosomal degradative enzymes (also called acid hydrolases) may roughly be classified into substrate-specific groups such as glycosidases, proteases, nucleases, lipases, phosphatases, phospholipases, and sulfatases, which altogether break down various biological substances including glycans, lipids, glycogen, and proteins. Lysosomal ions and ion channels are indispensable for the regulation of the lysosomal pH, and of the ion flux or transport across the lysosomal membranes (Xiong and Zhu 2016).

**Fig. 2:**
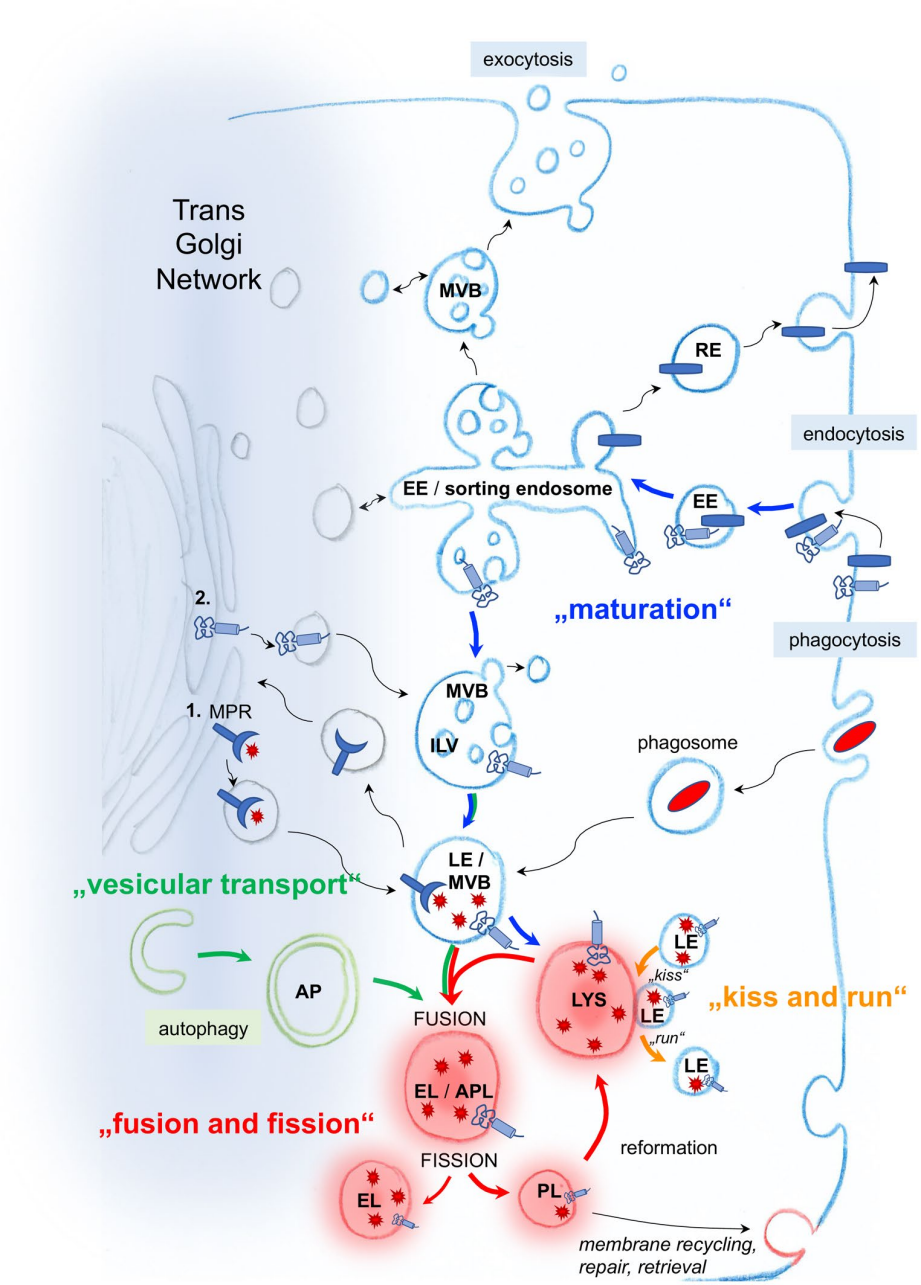
Lysosomal biogenesis is thought to occur through four different routes. In the *maturation pathway*, endocytosed cargo is first delivered to early endosomes, which progressively mature to late endosomes, endo-lysosomes, and subsequently into lysosomes. Thereby, endocytosis starts with the invagination of cargo-bound plasma membrane by either clathrin-dependent or clathrin-independent mechanisms. In the *vesicular transport pathway*, endosomal carrier vesicles/multivesicular bodies (MVBs) transfer cargo from early to late endosomes to lysosomes or directly from the matured late endosomes to lysosomes. In the *fusion and fission pathway*, the limiting membrane of late endosomes/MVBs and lysosomes fuses to form hybrid organelles, where the degradation of endocytosed macromolecules commences. These hybrid organelles subsequently reform to lysosomes. In the *“kiss and run” pathway*, late endosomes form a contact site (“kiss”) with lysosomes, transfer cargo and subsequently dissociate (“run”) again. Resident lysosomal proteins are synthesized in the ER. Sorting to lysosomes occurs through multiple pathways. 1. Most acid hydrolases are delivered to lysosomes via a mannose 6-phosphate (M6P) dependent ER-to-lysosome trafficking pathway. M6P modified proteins are recognized by M6P receptors (MPRs) in the trans-Golgi network (TGN), which forms small vesicles that carry lysosomal resident proteins. These vesicles fuse with late endosomes, the acidic environment of which causes dissociation of MPR from the cargos. While the MPRs recycle back to the TGN, lysosomal proteins continue their journey to lysosomes. 2. Lysosomal transmembrane proteins are either sorted directly from the ER-TGN to endosomes and lysosomes or indirectly, i.e., they are first sorted to the cell surface from where they enter the endocytic pathway. Both these direct and indirect routes rely on clathrin-coated vesicles to carry the proteins from the TGN or plasma membrane to the endosomes. Abbreviations: EE = early endosome, RE = recycling endosome, MVB = multi vesicular body, ILV = intraluminal vesicle, LE = late endosome, AP = autophagosome, LYS = lysosome, EL = endolysosome, APL = autophagolysosome, PL = protolysosome, MPR = mannose-6-phosphate receptor, light blue cylinder = lysosomal membrane protein, red star = lysosomal acid hydrolase, blue rod = plasma membrane protein, red oval = bacteria

Lysosomal biogenesis involves maturation from other endocytic vesicles (early endosomes, late endosomes/multivesicular bodies, and intermediate vesicles) and targeting of lysosomal proteins to the lysosome. Basically, four different routes to lysosome formation have been described (reviewed in (Trivedi et al. 2020)). These include a “maturation,” a “vesicular transport,” a “fusion and fission,” and a “kiss and run” pathway (Fig. 2). The large arsenal of acid hydrolases, transmembrane proteins, and membrane-associated proteins are specifically recognized as lysosomal components postsynthesis and thereafter correctly sorted to lysosomes. The vesicular pathway is used for the transport of the majority of lysosome resident proteins to lysosomes. Lysosomal transmembrane proteins are synthesized in the endoplasmic reticulum (ER). After passage through the Golgi apparatus, they are either sorted directly from the trans-Golgi network (TGN) to the endosomes and lysosomes or indirectly, i.e., they are first sorted to the cell surface from where they enter the endocytic pathway. Most acid hydrolases are delivered to lysosomes via a mannose 6-phosphate (M6P)-dependent ER-to-lysosome trafficking pathway (Griffiths et al. 1988) following their synthesis in the ER. M6P allows enzymes to be recognized by M6P receptors (MPRs) in the TGN, which forms small vesicles that carry lysosomal resident proteins. These vesicles fuse with late endosomes/multivesicular bodies (MVBs), whose acidic environment causes dissociation of MPR from the cargos. While the MPRs recycle back to the TGN, lysosomal proteins continue their journey to lysosomes (Ghosh et al. 2003). Lysosomes can therefore be distinguished from endosomes due to their lack of MPR. Other soluble enzymes and nonenzymatic proteins are transported to lysosomes in an M6P-independent manner mediated by alternative receptors such as the lysosomal integral membrane protein LIMP-2 or sortilin (Reczek et al. 2007; Zhao et al. 2014).

Endocytosed cargo can escape degradation via recycling vesicles. One of the best understood recycling process at lysosomes/late endosomes is the cellular iron metabolism and iron recycling (reviewed in (Kurz et al. 2011))*.* Regeneration of consumed lysosomes occurs in a process called autophagic lysosome regeneration (ALR) (Yu et al. 2010). There, proto-lysosomes bud-off from autophagolysosomes through extrusion of tubular structures composed of lysosomal membrane components. Proto-lysosomes mature to nascent lysosomes by acquiring acidity and lysosomal luminal proteins (Chen and Yu 2017). Further, lysosomes are regenerated from hybrid organelles (endolysosomes), which are generated by fusion of late endosomes with lysosomes to (Huotari and Helenius 2011).

## Cellular functions of lysosomes

**Fig. 3:**
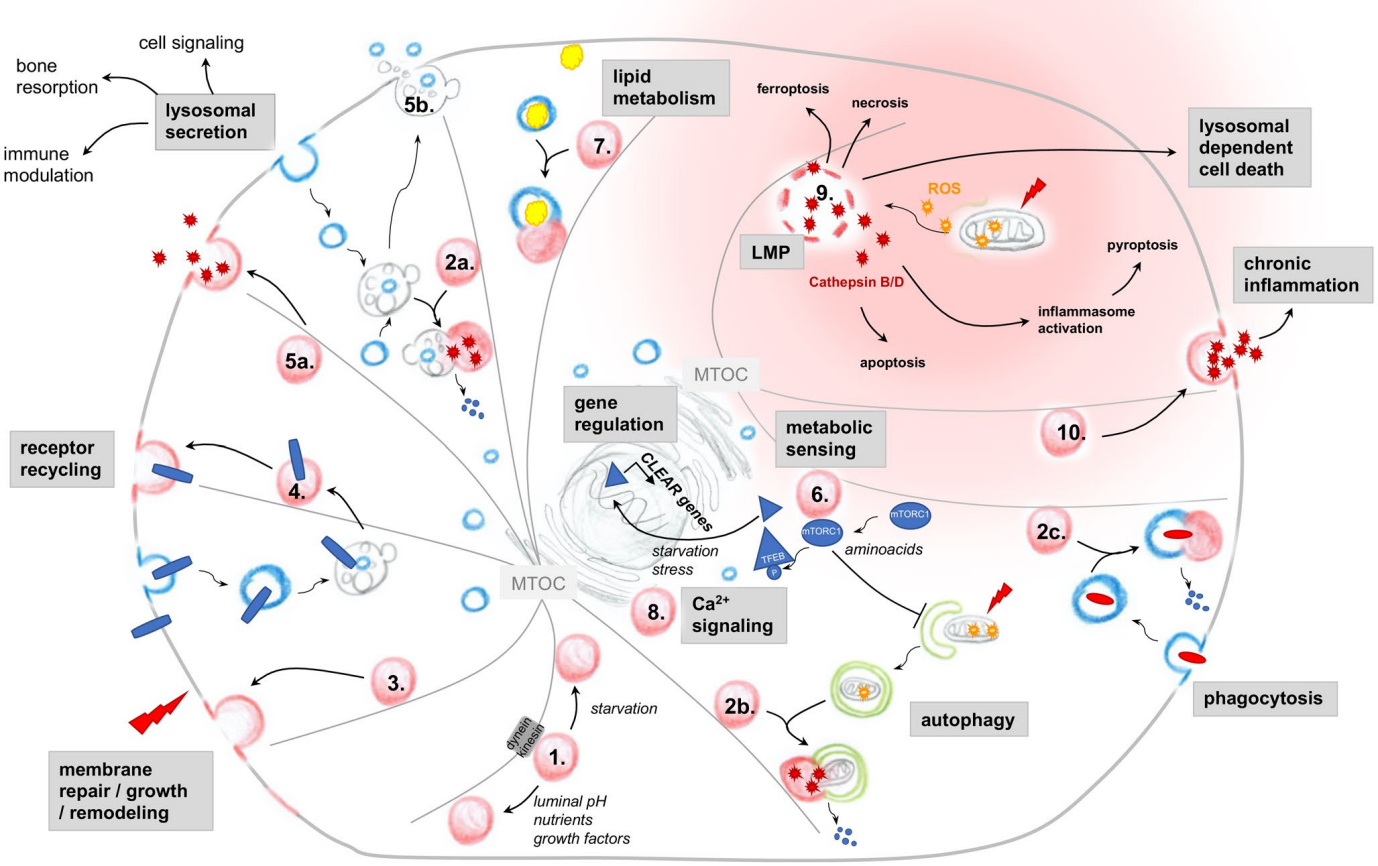
Scheme depicting the plethora of lysosomal functions within a cell. 1. Depending on the trigger, lysosomes move with the help of a dynein/kinesin motor complex along microfilaments that are organized by the microtubule organizing center (MTOC) and localize in the cell periphery or at the cell center. Cell-homeostatic functions of lysosomes include 2. degradation of cargo delivered from the 2a. endocytic, 2b. autophagosomal and 2c. phagocytic pathways to peptides, 3. membrane repair, remodeling and growth, 4. receptor recycling, 5. bone resorption, immune modulation and cell–cell signaling through lysosomal secretion of 5a. enzymes or 5b. exosomes derived from multivesicular bodies, 6. metabolic sensing and control through autophagy and gene transcription regulation, 7. lipid metabolism, and 8. inter-organelle Ca^2+^ signaling. Lysosomes can induce chronic inflammation and trigger diverse sorts of cell-deaths by 9. lysosomal membrane permeabilization (LMP) and 10. lysosomal secretion of pro-inflammatory factors. LMP is most frequently the consequence of reactive oxygen species (ROS) exposure (i.e., released from damaged mitochondria), which results in the leakage of lysosomal enzymes to the cytoplasm, inducing proinflammatory events such as inflammasome activation. Lysosomal dependent cell death is the consequence of massive lysosomal enzyme leakage

Lysosomes are involved in a multitude of cellular functions by far exceeding their role as degradative organelles. The most important lysosomal functions, namely exocytosis of proteins and of vesicles, plasma membrane repair, remodeling and growth, inter-organelle and inter-cellular signaling, metabolic sensing, lipid metabolism, and cell injury are discussed in the following paragraphs and summarized in Fig. 3.

### Protein exocytosis

Two types of lysosomes are described to exist physiologically, namely conventional degradative lysosomes and secretory lysosomes. Whereas conventional lysosomes primarily act as a waste disposal of phagocytosed and autophagocytosed debris (Klionsky and Emr 2000; Stuart and Ezekowitz 2005), the so-called *secretory lysosomes* (or lysosome-related organelles (LROs)) store and secrete proteins for cell-type-specific functions. Typical proteins secreted by lysosomes are lysosomal hydrolases, perforin, granzyme, histamine, serotonin, major basic protein, chemoattractant, clotting factors, and renin among others. The existence of secretory lysosomes is not restricted to a specific cell type anymore, as they have been described in hematopoietic cells, melanocytes, renal tubular cells, osteoclasts, and melanocytes (Blott and Griffiths 2002; Dell'Angelica et al. 2000; Lee and Ye 2018; Taugner et al. 1985). Some LROs are simply modified lysosomes, for instance in cytotoxic T lymphocytes, the lytic granules are the only lysosome-type organelle present. In other cells, such as melanocytes, both the melanosomes and the “conventional” lysosomes co-exist (Pryor and Luzio 2009). It is now widely accepted that secretory lysosomes move towards the plasma membrane, fuse with the plasma membrane and release their content into the extracellular space by exocytosis (Buratta et al. 2020). The exocytosis of lysosomal proteins is a ubiquitous Ca^2+^ regulated mechanism, which plays a crucial role in several physiological processes such as bone resorption by osteoclasts (Vaes 1968), melanocyte function during pigmentation, immune response and antigen presentation among others (Blott and Griffiths 2002; Buratta et al. 2020; Lee and Ye 2018).

### Vesicle exocytosis

Besides the exocytosis of proteins from secretory lysosomes, “lysosomes” have been shown to exocytose small extracellular vesicles (EVs), termed exosomes from cells. EVs are now classified into 3 distinct populations including apoptotic bodies, microvesicles, and exosomes (Thery et al. 2018). Among them, exosomes represent the smallest class of EVs (approximately 50–140 nm, despite no clear cutoff size to separate them from microvesicles) that originate from the inward budding of the membrane of late endosomes/lysosomes. Exosomes do not fuse with the late endosomal/lysosomal membrane but are released upon exocytosis as intact vesicles into the extracellular space and fluids (i.e. blood, synovial fluid, cerebrospinal liquor, and urine) (Buratta et al. 2020). In nonsecretory cells, this mode of exocytosis depends on membrane proximal lysosomes (Jaiswal et al. 2002). The process of *exosome exocytosis* is an important mechanism of cellular clearance, now also being more and more considered as an effective cell-to-cell communication mechanism, short and over long distance through fusion of exosomes with the membrane of other (neighboring) cells. Exosomes contain a plethora of cellar components from the donator cell, ranging from proteins, lipids, microRNA, RNA, and even DNA (Skotland et al. 2017). Interestingly, lysosomes have been suggested to restrict exosome exocytosis. In this process, MVBs were described to fuse to autophagosomes to form amphisomes. Amphisomes then fuse to lysosomes, which determine MVB fate and thereby reduce exosome release (Baixauli et al. 2014). Precisely, reduction of exosome release was found to occur in the setting of enhanced autophagy and enhanced LC3 levels (Fader et al. 2008).

### Plasma membrane repair, remodeling, and growth

Plasma membrane disruption is a normal event in the life of a mammalian cell, especially in cells exposed to mechanical stress. Resealing is therefore a life assuring event for irreplaceable cells (i.e., cardiac myocytes and neurons) (McNeil and Kirchhausen 2005). Lysosome exocytosis has been found to be essential for the repair of damaged plasma membrane via fusion of the lysosomes situated in proximity of the injury site with the plasma membrane (McNeil and Kirchhausen 2005; Reddy et al. 2001). Mechanistically, this process is driven by a transient increase in intracellular Ca^2+^ and the Ca^2+^ sensing membrane protein synaptogamin VII, which associates with lysosomes by interacting with the SNARE complexes and plasma membrane phospholipids (Caler et al. 2001). Whether the fusion of lysosomal membrane with the plasma membrane is accompanied with the exocytosis of proteins or vesicles, or whether the fusing lysosomes are those originating from lysosomal recycling events is not known. However, the process of lysosomal exocytosis as a membrane donator is not only important for plasma membrane repair but also for specific membrane remodeling mechanisms (Czibener et al. 2006) and for the elongation of developing neuronal processes (Arantes and Andrews 2006).

### Lysosomes as signaling nodes

Lysosomes can modulate cellular signaling through two principles: first, through their involvement in the recycling of receptors to the plasma membrane by the endocytic pathway; second, lysosomes have emerged as the second largest intracellular storage organelle for Ca^2+^, a ubiquitous primary signaling messenger in mammalian cells. Lysosomes typically comprise 2–3% of the cellular volume in mammalian cells, which suggests that although lysosomes have a high intraluminal Ca^2+^ concentration ([Ca^2+^]_LYS_ ≈ 500–600 µM, which is about ~ 5000–6000 times the amount of free Ca^2+^ compared to the cytosol (Lloyd-Evans et al. 2010)), their overall contribution to cellular Ca^2+^ signaling is relatively low compared to the ER, which comprises about 11% of cellular volume and has an approximate Ca^2+^ concentration of [Ca^2+^]_ER_ ≈ 12–2000 µM. Nonetheless, lysosomes have been shown to initiate local and global changes in cellular Ca^2+^ signaling. Local Ca^2+^ changes in the vicinity of lysosomes (through release of Ca^2+^ from the lysosome lumen) governs lysosomal transport and fusion events. Global Ca^2+^ release events appear to depend on the interplay between lysosomes and the ER. Thereby, a local lysosomal Ca^2+^ release at ER-lysosomal contact sites is thought to evolve into global Ca^2+^ release events. Further, ER-lysosomal contact might provide a source to refill depleted lysosomal Ca^2+^. Three main types of Ca^2+^ channels have been identified in mammalian lysosomes: transient receptor potential cation channels of the mucolipin family (TRPML), two-pore channels (TPR), and the trimeric Ca^2+^ two-transmembrane channel P2X_4_ (Li et al. 2019; Morgan et al. 2011). Lysosomal Ca^2+^ channel activities are most likely differentially modulated depending on the cell condition, thus allowing selective Ca^2+^ responses tailored to the cellular need, as they respond to a variety of stimuli, such as pH, nutrients, cellular stress, ATP, NAADP, and sphingosine. No actual candidate transporters or pumps have yet been identified in placental mammals that mediate the Ca^2+^ import into lysosomes necessary to obtain the high [Ca^2+^]_LYS_, however, SERCA3 (Lopez et al. 2008) and SLC24A5 (Na^+^/K^+^/Ca^2+^ exchanger (Lamason et al. 2005)) might represent potential candidates.

### Lysosomes as metabolic sensors

Being the main mediator of cellular catabolism, the lysosome is in a unique position to integrate information on cellular degradation and recycling processes as a proxy to sense the cell’s nutritional status (Saftig and Puertollano 2020). The nutrient-regulated “mechanistic target of rapamycin complex 1” (mTORC1), a key regulator of cellular biosynthesis pathways (Saxton and Sabatini 2017), dynamically associates with and is activated at lysosomes under specific conditions, especially in response to amino acids through involvement of heterodimeric Rag GTPases (RagA or RagB with RagC or RagD) at the lysosomal limiting membrane (reviewed in Ballabio and Bonifacino 2020; Lawrence and Zoncu 2019). mTORC1 recruitment is also inducible by cholesterol through involvement of the cholesterol-binding Niemann-Pick type C1 protein (NPC1). Activation of mTORC1 in response to nutrient availability increases the peripheral appearance of lysosomes to support cell anabolism and growth on the one side. On the other side, activation of mTORC1 inhibits catabolic pathways such as autophagy, which is visible in an inhibited fusion of lysosomes with autophagosomes. Of note, mTORC1 also regulates the re-formation of lysosomes during autophagy, a process that results in the restoration of a pool of functional lysosomes, which will be needed in the setting of starvation (Yu et al. 2010). mTORC1 is not the only nutrient-responsive molecule recruited to lysosomes. Rag-GTPases also mediate the recruitment of TSC (tuberous sclerosis complex) and FNIP (folliculin interacting protein 1) complexes, which both represent upstream regulators of mTOR.

Lysosomes sense the cellular nutrition state and transfer this information into an adaptive transcription of genes involved in several steps of the lysosome-autophagy pathway (Palmieri et al. 2011). This lysosome-to-nucleus signaling pathway is mediated through calcium signaling molecules such as transcription factor EB (TFEB) and the phosphatase calcineurin. TFEB dephosphorylation through calcineurin and protein phosphatase 2A (PPA2) (Martina and Puertollano 2018; Medina et al. 2015) mediates its translocation from the lysosomal limiting membrane to the nucleus. There, TFEB upregulates the transcription of CLEAR genes, such as hydrolases, lysosomal membrane proteins, the v-ATPase proton pump complex, and participates in the regulation of lysosome-related processes such as endocytosis, exocytosis, phagocytosis, and immune response (Sardiello et al. 2009). TFEB simultaneously controls the expression of autophagy genes and by this regulates both the initiation and the terminal steps of this essential homeostatic pathway (Raben and Puertollano 2016). TFEB nuclear translocation is promoted by a multitude of environmental stimuli, including starvation, bacterial infections, physical exercise, ER stress, oxidative stress, and mitochondrial damage. Transcription of autophagosomal-lysosomal genes is shut-off by the nuclear export and cytosolic retention of TFEB, which is regulated by its phosphorylation through the kinases mTORC1, and potentially also by Erk and glycogen synthase kinase 3 (GSK3) (Li et al. 2018, 2016; Settembre et al. 2011).

Besides the capability of sensing the cells nutritional/metabolic status, lysosomes exhibit a cargo recognition response. In this response, lysosomes can sense the arrival of autophagosomes carrying damaged mitochondria or microbes by recognizing mitochondrial or microbial nucleic acid via toll-like receptors (TLRs) expressed at the lysosomal limiting membrane. Of the thirteen members of the TLR family, TLR3, TLR7/8, and TLR9 signal from endolysosomes. When activated by nucleic acids, these receptors initiate a proinflammatory signaling cascade through nuclear translocation of NF-κB or interferon regulatory factors to stimulate the production of cytokines and/or interferons. TLR9 dow nstream signaling events are further an important mechanism to adapt the degradative potential of a cell to the autophagic cargo (Ballabio and Bonifacino 2020).

### Lysosomes in lipid metabolism

In line with their digestive function, lysosomes among other vesicles stand at the crossroad of lipid metabolism due to their ability to process and sort exogenous and endogenous lipids to various membrane compartments (reviewed in (Thelen and Zoncu 2017)). Exogenous sterols, triglycerides, and phospholipids, which are carried by LDL enter the cell via receptor-mediated endocytosis and are processed by the lysosome (Goldstein and Brown 2015). Lysosomes also sort and recycle endogenous lipids, which have entered lysosomes through fusion (i.e., inner membrane of autophagosomes) or maturation (i.e., limiting membrane of intra luminal vesicles (ILVs) from MVBs). These intralysosomal membranes coalesce into membrane sheets or “whorls,” as their lipid composition is modified in the internal acidic environment of the lysosome (Schoneberg et al. 2017). They need to be removed from the lysosomal lumen and are inserted into the lysosomal membrane to be further delivered to other organelles by vesicular routes. One such receiving organelle is the ER, where the lipids may be esterified and stored in lipid droplets. Once within lysosomes, taken up LDL particles are attacked by the lysosomal acid lipase type A (LIPA), which de-esterifies the cholesterol and triglyceride molecules (Chang et al. 2006). Because the level of cholesterol in the limiting membrane of lysosomes is low (Kolter and Sandhoff 2005), a transport process across this membrane followed by delivery to another membrane is thought to exist. Export of cholesterol from lysosomes his mainly linked to the concerted action of Niemann-Pick type C (NPC) 1 and 2 (Infante et al. 2008) in parallel to LIMP2 (Heybrock et al. 2019). Possibly, LAMP1 and LAMP2 accept cholesterol from NPC2 to facilitate the flow of cholesterol (Li and Pfeffer 2016). Once cholesterol is deposited on the lysosomal membrane, its transport to extra-lysosomal locations is thought to occur rapidly.

Lysosomal lipid metabolism is directly synchronized to the cellular needs via mTORC1-TFEB pathway, which promotes de novo lipid synthesis and simultaneously suppresses lipid catabolism. Involved mechanisms for enhanced lipid synthesis are the mTORC1-dependent phosphorylation of S6 kinase 1 (S6K1) (Duvel et al. 2010) and the mTORC1-driven expression, trafficking and proteolytic processing of sterol-responsive element-binding proteins (SREBPs) SREBP1c and SREBP2, which are master regulators for the synthesis of fatty acids and sterols respectively (Thelen and Zoncu 2017). Lysosomal lipid catabolism is prevented by the mTORC1-TFEB pathway by inhibiting the breakdown of lipid droplets in a specialized autophagosomal process called lipophagy.

### Lysosomal injury

Lysosomes exert a cytoprotective effect by removing damaged organelles such as mitochondria, which are delivered to lysosomes by the autophagosomal pathway. As damaged organelles release pro-apoptotic proteins, their removal protects from cell injury (Ravikumar et al. 2010). On the other side, most lysosomes contain relatively large amounts of redox-active iron and in combination with a lack of antioxidant enzymes are therefore unusually susceptible to oxidant-mediated membrane destabilization or rupture, which perpetuates cell injury (Kurz et al. 2008). Features of lysosomal dysfunction include changes in expression and/or activity of lysosomal enzymes, changes in lysosomal size/number/pH/cellular positioning/motility and changes in lysosomal membrane properties. Furthermore, lysosomal dysfunction can lead to the accumulation of undegraded material and give rise to blockade of lysosomal related pathways (especially autophagy). Lysosomal damage leads to a condition called lysosomal membrane permeabilization (LMP). This selective destabilization of the lysosomal membrane allows translocation of lysosomal contents, including ions and cathepsins to the cytoplasm through membrane ruptures. To cope with lysosomal injury, cells have developed “endolysosomal damage-response mechanisms,” of which 4 have been described to date: (1) creation of new lysosomes via the mTORC1-TFEB pathway; (2) repair of damaged lysosomes by membrane sealing; and removal of damaged lysosomes either by (3) lysophagy; and/or by (4) lysosome exocytosis (Samie and Xu 2014). Small (proton permeable) disruptions of the lysosomal limiting membrane activate lysosomal repair mechanisms which converge at an ESCRT-dependent resealing of the membrane (Skowyra et al. 2018). Strong ruptures of the lysosomal membrane (i.e., ruptures permeable to lysosomal proteins, which are then exposed at the cytosolic side of the lysosomal membrane, such as β-galactosidase) trigger the recruitment of cytosolic galectins to injured lysosomes. Galectins (Chauhan et al. 2016; Thurston et al. 2012) together with extensive lysosomal ubiquitination (Papadopoulos and Meyer 2017) recruit components of the autophagy machinery and thus orchestrate the removal of damaged lysosomes with their debris by a selective form of autophagy, a process called lysophagy (Maejima et al. 2013).

Massive and prolonged LMP leads to a special form of programmed cell death, called lysosome-dependent cell death (LDCD), of which the extruding cathepsin B and cathepsin D are the main executors (reviewed in (Wang et al. 2018)). These through LMP extruding proteases are thought to further proteolytically activate substrates such as Bid and Bax, which in turn promote mitochondrial outer membrane permeabilization (MOMP) and caspase activation resulting in the initiation of apoptosis (de Castro et al. 2016). In other cases, the release of lysosomal effectors through LMP can result in two other forms of cell death depending on the triggering effector, namely ferroptosis (iron as trigger), and pyroptosis (reactive oxygen species as trigger). Last but not least, total lysosomal rupture leads to the massive release of lysosomal enzymes and thereby to an uncontrolled cleavage of cellular components and cytosolic acidification culminating in necrosis. Lysosomal injury/impairment has been implicated in various disease conditions including lysosomal storage diseases, neurodegeneration, autoimmune diseases, and cancer.

## Lysosomes in glomerular health

The kidney glomerular filtration barrier represents a sophisticated syncytium of three individual cell types, namely podocytes, mesangial, and glomerular endothelial cells, which sustain the structure and regulate the function of the filtration barrier (Pavenstadt et al. 2003). The terminally differentiated podocytes embrace the glomerular capillaries and form an intricate mesh with their interdigitating processes that are interconnected by a modified form of adherens junction, the slit diaphragm, which ultimately bridges the filtration slits. Podocytes are thought to share many morphological features and proteins with neurons (Giardino et al. 2009; Rastaldi et al. 2003). The intraglomerular mesangial cells are situated in close contact with the endothelial cells and represent a specialized form of pericytes that provide structural support. The fenestrated endothelial cells line the glomerular capillaries and reside opposite from the podocytes separated by the glomerular basement membrane. Podocytes synthesize, maintain, stabilize the mature glomerular filtration barrier, and regulate glomerular filtration. Mesangial cells regulate the glomerular capillary flow and ultrafiltration surface, are responsible for the homeostasis of the mesangial matrix and are source and targets of growth factors, cytokines, and vasoactive agents. Further, they are phagocytic cells degrading glomerular basal lamina and immune complexes. Glomerular endothelial cells are involved in GBM production and contribute the hydraulic conductivity and size and charge selectivity of the glomerular filtration barrier (reviewed in (Meyer-Schwesinger, C., Huber, T.B. 2020). Malfunction of any of these cell types leads to loss of glomerular function and proteinuria leading to the concept that these glomerular cell types interact (Dimke et al. 2015).

### Lysosomes in healthy glomerular cells

In comparison to the surrounding renal tubular system the glomerular compartment is less abundant in lysosomes and shows lower lysosomal activity (Lovett et al. 1982). Knowledge about the glomerular cell-type “inventory” of lysosome types is rudimentary to absent. Electron microscopic evaluations demonstrate that the podocyte cell body contains prominent lysosomes. The density of cell organelles in the podocyte cell body indicates a high level of anabolic as well as catabolic activity. In contrast to the cell body, podocyte processes contain only few organelles (Pavenstadt et al. 2003), which distinguishes them from neuronal processes. Mesangial cells are phagocytic cells (Gomez-Guerrero et al. 2002; Schlondorff 1987), and therefore should contain an evolved lysosomal system. As a pioneer in lysosomal discovery, the group of George Palade was the first to describe the occurrence of an enhanced lytic activity in mesangial cells in comparison to glomerular endothelial cells and podocytes (Miller and Palade 1964). The measurement of acidic lysosomal hydrolases (acid phosphatase, β-glucuronidase, cathepsin D, nonspecific esterase, and aryl sulfatases A and B) showed considerable lysosomal activities in the nonpodocyte fraction of glomeruli (Lovett et al. 1982; Yokota et al. 1985). Besides the abundance of lysosomal hydrolases in mesangial cells, the morphological dissection of the mesangial and glomerular endothelial cell lysosomal system has largely been neglected.

### Glomerular cell-type-specific dependency of homeostatic degradation systems

One important feature has to be taken into consideration prior to reflecting about the individual contribution of lysosomes to the homeostasis of glomerular cell types. Whereas lipid catabolism is restricted to lysosomes, protein degradation is not solely achieved by lysosomes. In fact, the majority (about 80%) of cellular proteins are degraded by the ubiquitin proteasomal system (UPS), which interacts at multiple steps with the autophagosomal lysosomal pathway (Meyer-Schwesinger 2019). While lysosomes mostly degrade protein aggregates and membrane proteins delivered by the endocytic pathway, the proteasome is essential for the regulation of various cellular functions by degradation of mostly short-lived and regulatory proteins, or damaged and misfolded proteins (Meyer-Schwesinger 2019), and protein aggregates in case of lysosomal dysfunction (Hao et al. 2013; Hjerpe et al. 2016). Both degradation systems mutually compensate for the impairment of one another (Meyer-Schwesinger 2019). This can be nicely observed in a study, in which podocytes deficient for ATG5 (essential for autophagosome formation) compensate autophagosome deficiency by upregulation of the UPS (Hartleben et al. 2010), or in mice with the lysosomal storage disease mucolipidosis type III, where the glomerular UPS compensates for lysosome-associated degradation defects resulting from lysosomal enzyme missorting into the extracellular space (Sachs et al. 2020). Only upon impairment of the proteasome in aged mice do ubiquitinated nondegraded proteins accumulate, and does mild proteinuria occur in ATG5-deficient podocytes (Hartleben et al. 2010). Interestingly, mucolipidosis type III mice do not develop proteinuria with age, even though a multitude of lysosomal hydrolases are missorted and do not reach the lysosome (Sachs et al. 2020).

It is becoming more and more clear that glomerular cell types differentially depend on protein degradation systems. Of the three glomerular cell types, most research attention to this topic is centered around podocytes. Proteomic studies performed in cultured murine and human podocytes demonstrate a strong proteolytic shift from a predominant proteasomal activity to a lysosomal activity in the course of podocyte differentiation (Rinschen et al. 2016; Schroeter et al. 2018), which coincides with a globally increased stability of mitochondrial, cytoskeletal, and membrane proteins in differentiated podocytes (Schroeter et al. 2018). How far this finding can be transferred to the physiologic in vivo situation still needs to be determined, as possible skewers such as culture conditions could explain the proteolytic system shift from undifferentiated to differentiated podocytes. Nonetheless, the specific disruption of players of the endocytic system in podocytes using genetic approaches support a strong involvement of the lysosomes in podocyte health. Thereby, mice with a podocyte-specific deficiency of Vps34, which in yeast is essential for the sorting of hydrolases to yeast vacuoles (Herman and Emr 1990) and in mammals (as mVps34 (class III PI3K)) has been implicated in the regulation of autophagy (Itakura et al. 2008), develop severe glomerulosclerosis with enlarged vacuoles and increased autophagosomes in podocytes and die at 9 weeks of age (Chen et al. 2013). This phenotype appears to be the result of a direct lysosomal impairment rather than of a primary defect in autophagy, highlighting the complexity of the subject and the need for cell-type-specific investigations.

Dissection of the expression of lysosomal membrane proteins among glomerular cell types suggests the existence of different subsets of endocytic/lysosomal vesicles within podocytes, mesangial and glomerular endothelial cells. Thereby, Limp2-positive vesicles are predominantly found in mesangial and glomerular endothelial cells, whereas Lamp2 is evenly distributed within the glomerular cell types, however to a far lesser extent than in the surrounding parietal epithelial cells and proximal tubular cells (Sachs et al. 2020), Fig. 4a. The functional significance of this finding still needs to be evaluated. Recent data from our lab demonstrate that of all glomerular cell types, podocytes have an exceptional high dependence on a functioning proteasomal system in vivo. Thereby, a new technique enabling a reporter-free bulk separation of glomerular cell types from wild-type mice for protein biochemical investigations shows that podocytes express higher levels of proteasomal proteins in comparison to mesangial and glomerular endothelial cells, whereas lysosomal proteins are comparable between glomerular cell types (https://biorxiv.org/cgi/content/short/2020.08.22.262584v1).

**Fig. 4:**
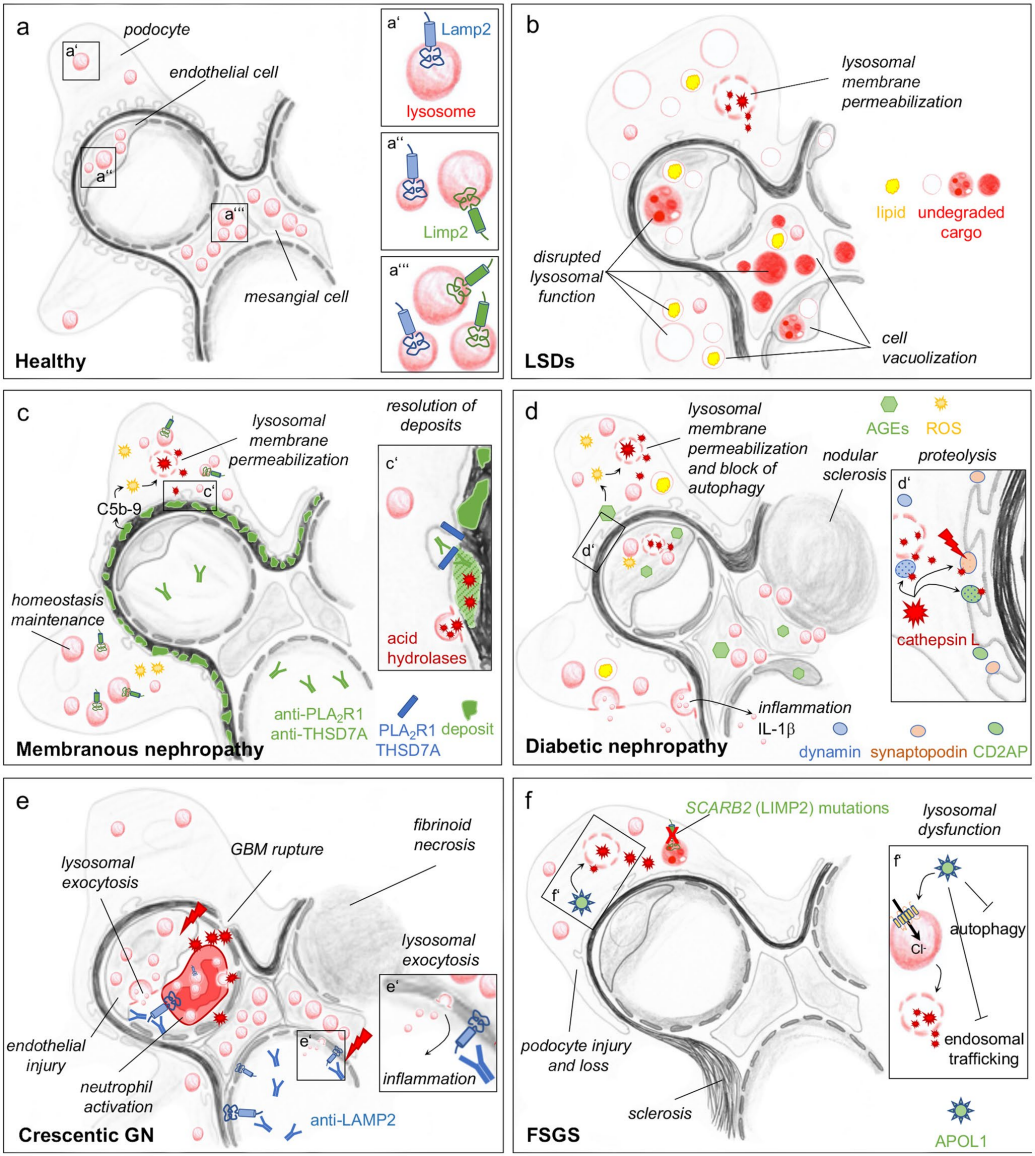
Scheme of glomerular cell involvement of lysosomes in health and disease. (a) Normal lysosomal distribution within glomerular cell types. (b) Lysosomal storage disorders (LSDs) with different kinds of accumulating undegraded lysosomal cargo, alteration of lysosomal morphology and function. (c) Membranous nephropathy is caused by the binding of autoantibodies directed against podocyte foot process proteins PLA_2_R1 and THSD7A. Subepithelial immune deposits and complement activation are thought to mediate podocyte injury. The membrane attack complex C5b-9 triggers ROS production and LMP. Lysosomal exocytosis of acid hydrolases is thought to mediate slow degradation of subepithelial deposits (c’). A specific upregulation of LIMP2-positive lysosomes occurs in MN, potentially to compensate for the impairment of proteasomal dysfunction. (d) Diabetic nephropathy with involvement of all three glomerular cell types. Advanced glycation end products (AGEs) induce the production of reactive oxygen species (ROS), which trigger lysosomal membrane permeabilization (LMP) with the sequel of acid hydrolase release such as cathepsin L into the cytoplasm. Cathepsin L cleaves podocyte proteins synaptopodin, CD2AP, and dynamin, inducing cytoskeletal alterations (d’). Inflammasome activation and lysosomal exocytosis of exosomes and of IL-1 β is additionally thought to maintain a pro-inflammatory condition. (e) Autoantibodies to LAMP2 have been described in crescentic glomerulonephritis (GN). These autoantibodies bind at the plasma membrane and at the limiting membrane of myeloperoxidase (MPO) and proteinase 3 (PR3) containing lysosomes in neutrophils and bind to a structurally related membrane protein at the surface of glomerular and renal microvascular endothelial cells. Lysosomal exocytosis (e’) is thought to be a major contributor to inflammation. (f) Focal segmental glomerulosclerosis (FSGS) can be the cause of mutations of SCARB2, encoding for LIMP2 in primary FSGS. Further, increased susceptibility of African Americans to FSGS have implicated variants of apolipoprotein L1 (APOL1) to lysosomal dysfunction in podocytes. APOL1 can induce LMP and a continuous chloride influx (f’). The APOL1 risk-variants interfere with endosomal trafficking and block autophagic flux, ultimately leading to inflammatory-mediated podocyte death and glomerular scarring

## Lessons learnt from lysosomal storage diseases

The role of lysosomes in glomerular cells can best be appreciated by analyzing the effects of primary lysosomal dysfunctions on the glomerulus. Lysosomal storage disorders (LSDs) are a group of individually inherited metabolic disorders that are the most common cause of childhood neurodegeneration. These diseases are predominantly caused by (1) mutations in genes encoding for lysosomal enzymes or their accessory proteins; (2) mutations in genes encoding for lysosomal transmembrane proteins that include transporters, ion channels, proteins of unknown functions (reviewed in (Cox and Cachon-Gonzalez 2012)); and (3) mutations in genes encoding for the Golgi-resident GlcNAc-1-phosphotransferase which tags lysosomal enzymes at the Golgi apparatus with M6P for proper trafficking to lysosomes (Kollmann et al. 2010; Velho et al. 2017). As different LSDs have different molecular causes, the effects on lysosomal storage and the (patho) biochemical consequences on lysosomal function strongly vary (Marques and Saftig 2019). Nonetheless, almost all associated diseases are characterized by similar changes in cell biology, which include intra-lysosomal accumulation of various substrates (lipids, proteins, gangliosides, glucosylceramides, glycogen, glycosaminoglycans, among others), defects in endocytosis, autophagy, and mitochondrial function (Cox and Cachon-Gonzalez 2012). These functional deficiencies impact multiple cell types, particularly neurons (resulting in neurodegenerative phenotypes), and in very rare cases glomerular cells.

### Glomerular involvement in LSD

The susceptibility of glomerular cells to lysosomal impairment is far from being clear, as it strongly varies between the different types of LSDs and also within one type of LSD. LSDs most likely affect glomerular cell types differentially due to a potentially differing capacity in lysophagy, ESCRT mediated membrane repair, lysosome mobility, exocytosis, cholesterol homeostasis among others (reviewed in (Marques and Saftig 2019)) (Fig. 4b). Renal/glomerular involvement constitutes a key contributor to the morbidity and mortality in patients with Fabry disease (Kantola 2019). In other LSDs, glomerular/renal impairment is usually a rare condition. Nonetheless, renal dysfunction has been reported in single patients with nephrosialidosis (Roth et al. 1988), infantile sialic acid storage disease (Sperl et al. 1990), cystinosis (Langman 2019), mucopolysacharidosis I (MPS-I) (Taylor et al. 1986), as well as with mucolipidosis (Kerr et al. 2011; Kobayashi et al. 2011; Tang et al. 1995). A complete review is beyond the scope of this article, a subset of LSDs with rare occurrence of glomerular cell involvement is summarized in Table 1. Although rapid progress has been made in understanding the genetic basis of lysosomal storage disorders, the factors underlying the disruption of cell metabolic and signaling pathways associated with these conditions are less well understood. It is commonly accepted that despite the broad variability of LSDs, diseases that store similar types of macromolecules (sphingolipids, mucopolysaccharides or glycoproteins) often have similar biochemical, pathological, and clinical phenotypes, suggesting that the nature and distribution of the stored material is the major lesion defining factor (Bifsha et al. 2007). In line, the electron density of the accumulated storage material differs between podocytes, glomerular endothelial and mesangial cells in mouse models of LSD (Sachs et al. 2020), suggesting that distinct types of macromolecules accumulate in a glomerular cell-specific manner, as the metabolic requirements might differ between the glomerular cell types. This, however, is pure speculation, as no information exists about the metabolic preferences of mesangial and glomerular endothelial cells. Murine podocytes have recently been shown to largely depend on anaerobic glycolysis (Brinkkoetter et al. 2019). Further, the nature of accumulating macromolecules underlying the different deposits by electron microscopy are mostly not determined in a glomerular cell type manner.

**Table 1:** Lysosomal storage disorders (LSDs) in which renal involvement has been reported. In the others renal involvement is based on 1 or more clinical reports

Disease (#OMIM)	Gene	Protein	Protein function	Renal findings	Citations
Cystinosis #219750 #219800	*CTNS*	Lysosomal membrane protein cystinosin	Cystine transport across the lysosomal membrane	Renal tubular Fanconi syndrome, renal failure, renal calculi	(Langman 2019)
Fabry disease #301500	*GLA*	Lysosomal enzyme α-Galactosidase A	Hydrolysis of the terminal alpha-galactosyl moieties from glycolipids and glycoproteins	Humans: Glomerular endothelial cells, hypertrophic foamy podocytes with zebra bodies, mesangial expansion	(Alroy et al. 2002; Kantola 2019)
Nephrosialidosis #256150	*NEU1*	Lysosomal enzyme α-(2–6) neuraminidase	Cleavage of glycosidic linkages of neuraminic acids	Frequently observed glomerulopathy that develops early and causes death. Diffuse and severe vacuolization of glomerular and tubular cells	(Roth et al. 1988) (Chen et al. 2011) (Maroteaux et al. 1978) (Maroofian et al. 2018) (Tylki-Szymanska et al. 1996)
Mucopolysaccharidosis I #607016	*IDUA*	Lysosomal enzyme α-L-iduronidase	Degradation of glycosaminoglycans	A rare case of nephrotic syndrome	(Clarke 1993; Moore et al. 2008; Taylor et al. 1986)
Mucolipidosis II/III #252500 #252605	*GNPTAB* *GNPTG*	Golgi-resident GlcNAc-1-phosphotransferase	M6P modification of mannose residues on lysosomal enzymes	Rare cases of foamy podocytes	(Kerr et al. 2011; Kobayashi et al. 2011; Tang et al. 1995)
Mucolipidosis type IV #252650	*TRPML1*	Lysosomal Ca^2+^ channel	Mediates Ca^2+^ release from the lysosomal lumen to the cytosol, can be activated by starvation, reactive oxygen species, Phosphatidylinositol 3,5 bisphosphate	Urine analysis showed accumulation of all phospholipid species, of several glycolipids, and of gangliosides	(Bargal et al. 2000) (Bassi et al. 2000) (Caimi et al. 1982)
Niemann-Pick #257200 #607616 #257220	*SMPD1* *NPC1* *NPC2*	Lysosomal enzyme acid sphingomyelinase (ASM) NPC1 and NPC2: lysosomal cholesterol binding proteins	ASM: breakdown of sphingomyelin to ceramide and phosphorylcholine NPC1 and NPC2: regulation of intracellular cholesterol trafficking	Rare cases of human sphingolipid accumulation in kidney and in SMPD1 knockout mice	(Briere et al. 1976; Kuemmel et al. 1997)
infantile sialic acid storage disease #269920	*SLC17A5*	Lysosomal vesicular excitatory amino acid transporter (VEAT)	H^+^-coupled sialic acid exporter in lysosomes	Nephrotic syndrome	(Sperl et al. 1990) (Miyaji et al. 2008) (Lemyre et al. 1999)
Gaucher disease #230800	*GBA*	Lysosomal enzyme β-Glucosidase	Cleavage of the β-glucosidic linkage of glycosylceramide	Mesangial and glomerular endothelial cells	(Boer et al. 2020) (Chander et al. 1979)
Sandhoff disease #268800	*HEXB*	Lysosomal enzyme β-subunit of hexosaminidase A	Breakdown of gangliosides	Very rare cases of kidney globosid accumulation	(Tatematsu et al. 1981)
Tay-Sachs disease #272800	*HEXA*	Lysosomal enzyme α-Subunit of hexosaminidase A	Breakdown of gangliosides	Very rare cases of kidney globosid accumulation	(Sandhoff et al. 1968)
Farber lipogranulomatosis #228000	*ASAH1*	Lysosomal enzyme Acid ceramidaseβ	Hydrolysis of ceramide into sphingosine and free fatty acid	Humans: One case of renal lipogranulomatosis, mice: nephrotic syndrome	(Devi et al. 2006; Li et al. 2020a; Samuelsson and Zetterstrom 1971; Yu et al. 2018)

### Phenotypic discrepancies between murine models of LSD and human disease-related phenotypes

The phenotypic heterogeneity associated with a given genotype in LSDs demonstrates that even though disease originates from a single gene disorder, other (cell-type specific?) modifying factors must play a role. One such modifying factor could be the compensatory potential of the affected cell species in dealing with this storage stress, which could define/shape the characteristics of the clinical presentation. Of all glomerular cells, podocytes have most frequently been observed to exhibit an altered morphology in lysosomal storage disorders, usually described as a foamy appearance (Kerr et al. 2011; Kobayashi et al. 2011; Tuysuz et al. 2016), most likely due to the accumulation of storage material. However, this foamy podocyte appearance does not necessarily associate with a decreased renal/glomerular function in for example mucolipidosis patients (Kerr et al. 2011). In this setting, differential “rescue” mechanisms are activated within glomerular cell types to compensate for lysosomal dysfunction. These rescue mechanisms range from an activation of lysosomal biogenesis, an activation of the proteasomal system, a downregulation of mTORC1 signaling, and a downregulation of protein synthesis through the integrated stress response (Sachs et al. 2020), among others. An important fact that has hampered the unraveling of the nature of glomerular cell injury in the different forms of LSDs is that often the experimental animal models of LSD do not mirror the human disease-related phenotypes despite the fact that they carry the human genotype. This limitation is most likely due to possible species-based differences in glomerular cell metabolism (Abe et al. 2000; Mattocks et al. 2006).

#### Fabry disease

A prominent example of species difference in dealing with the sequel of a defined genetic lysosomal disorder is Fabry disease, an X-linked monogenic LSD caused by mutations in the GLA gene (Kantola 2019), which encodes for the lysosomal hydrolase α-galactosidase A. The lack of α -galactosidase A leads to the progressive accumulation of glycosphingolipids, such as globotriaosylceramide (Gb3) in lysosomes of a plethora of cell types (mainly in endothelial cells of the kidney, heart liver, and spleen) as well as in plasma. Even though the glomerular/podocyte expression of α-galactosidase A in normal human kidneys is weak to absent (Christensen et al. 2007), renal and glomerular involvement is common in Fabry patients and constitutes a key contributor to the morbidity and mortality. In contrast, mouse models of Fabry disease fail to exhibit glomerular pathology (Ohshima et al. 1997), despite the comparable prevalence of glycosphingolipid storage per renal cell type including podocytes, and interstitial cells, vascular endothelial and smooth muscle cells, as well as the tubular cells of the distal tubules and loop of Henle (Kantola 2019). Gb3 accumulations significantly differ between humans and mice in mesangial (higher in humans) and in proximal tubular cells (higher in mice) (Valbuena et al. 2011). Another interesting finding concerning glomerular cell-type-specific differences in lysosomal dependency is the fact that in Fabry patient’s enzyme replacement therapy (ERT) with α-galactosidase A efficiently clears Gb3 accumulation in glomerular endothelial and mesangial cells, however, to a far lesser extent in podocytes (Thurberg et al. 2002). Human podocytes take up recombinant α-galactosidase A during ERT through the endocytic receptors MPR, sortilin, and megalin (Prabakaran et al. 2011) (of note: megalin expression in human podocyte is debated (Kerjaschki and Farquhar 1983)). The endocytosis activity for lysosomal acid hydrolases such as α-galactosidase A (Christensen et al. 2007) and arylsulfatase B (Hendrickx et al. 2020) seems, however, to be weaker in podocytes than in other (renal) cell types such as proximal tubular cells (Christensen et al. 2007). Nonetheless, podocyte injury and loss correlate with Gb3 accumulation (Najafian et al. 2020), suggesting that podocytes are especially sensitive to Gb3. Pathomechanistically, globotriaosylsphingosine (lyso-Gb3), which also accumulates in Fabry disease (Gold et al. 2013), promotes Notch1-mediated inflammatory and fibrogenic responses in podocytes that may contribute to Fabry nephropathy (Sanchez-Nino et al. 2015) among others.

#### SCARB2 mutations

One further example for species-related differences can be seen in humans with mutations in *SCARB2*, which encodes for LIMP2. Patients present with action myoclonus renal failure (AMRF) accompanied by storage in the brain but not in the kidney. Renal failure and proteinuria in humans with AMRF is thought to relate to hypertrophy and hyperplasia of podocytes in the early phase of injury and severe focal glomerulosclerosis with glomerular collapse as an endpoint (Berkovic et al. 2008). Limp2-deficient mice on the other hand present with obstruction of the ureteric pelvic junction, deafness and a peripheral demyelinating neuropathy and exhibit slight proteinuria with the occurrence of mesangial expansion, thickening of the glomerular basement membrane and foot process effacement, however, without the development of FSGS-like lesions (Berkovic et al. 2008; Desmond et al. 2011; Gamp et al. 2003).

#### Farber disease

A recent publication demonstrated that mice with a podocyte-specific deficiency of lysosomal acid ceramidase, exhibited podocyte injury due to the accumulation of ceramide and nephrotic syndrome (Li et al. 2020a). The related human genetic mutations leading to deficiency of lysosomal acid ceramidase lead to Farber disease, which is characterized by subcutaneous nodules, joint pain, and voice hoarseness. Patients also present with enlarged liver and spleen and with neurologic and respiratory complications. In this rare LSD, ceramidase activity has not been recognized in the kidney (Yu et al. 2018), but one case reports ceramide accumulation in the kidney (Samuelsson and Zetterstrom 1971). No description of ceramide accumulation in podocytes or nephrotic syndrome exist to date.

#### Gaucher disease

In the most common LSD, Gaucher disease, glomerular involvement is (again, as typical for LSD) extremely rare. However, seldom cases of glomerular glucocerebroside (Gaucher bodies) accumulation are described, mainly in glomerular endothelial and mesangial cells and are thought to be indicative of the phagocytic potential and of the deficiency of glucocerebroside-cleaving enzyme in these cells (Chander et al. 1979). Gaucher disease arises from an autosomal recessive deficiency of the lysosomal enzyme glucocerebrosidase (encoded by the *GBA* gene) resulting in a decreased lysosomal degradation of the sphingolipid glucocerebroside, which is a normal intermediate in the degradation of membrane compounds (Peters et al. 1977). Numerous mutations in the *GBA* gene have been associated with Gaucher disease whose hallmark are the Gaucher cells, lipid-laden macrophages with lysosomal glucocerebroside deposits (reviewed in (Boer et al. 2020)). Why glomerular cells, especially podocytes are so rarely affected in Gaucher disease, is not known, as more and more studies demonstrate the importance of a balanced lipid metabolism, especially of sphingolipids for podocytes (reviewed in (Merscher and Fornoni 2014)). Sphingolipids are involved in the formation of lipid rafts in podocytes and are essential for the maintenance of a functional slit diaphragm in the glomerulus. To different degrees, sphingolipids not only accumulate in glomerular cells in Gaucher disease but also in other LSDs such as Tay-Sachs, Sandhoff, Fabry, hereditary inclusion body myopathy 2, Niemann-Pick, and nephrotic syndrome of the Finnish type (Merscher and Fornoni 2014).

## Lysosomes in nonhereditary glomerular diseases

Our knowledge about a direct involvement of lysosomes to glomerular cell pathology in nonhereditary diseases is scarce. The typical morphologic alterations of increased lysosomal size and amount, which suggest an affected lysosomal function have been described in many contexts. However, whether the cause of increased lysosomal size/amount is an underlying direct lysosomal disturbance(s) or merely a compensatory upregulation of lysosomes to maintain cellular homeostasis remains largely unknown. Most research attention in glomerular injury has been given to autophagy. Autophagy is directly coupled to lysosomal function as one arm of the endocytic pathway but does not necessarily mirror lysosomal function in glomerular cells per se, as autophagy can be altered in a lysosome-independent way, i.e., in the case of failure to form autophagosomes (Hartleben et al. 2014). Therefore, this review will focus on those findings that directly examine lysosomal function in the context of glomerular cell injury. Many excellent reviews exist, which discuss the importance of autophagy and mTOR in podocyte (and to a lesser extent for glomerular) physiology and pathophysiology (Cui et al. 2020; Cybulsky 2017; Lenoir et al. 2016).

Sphingolipid accumulation, which frequently is related to disturbed lysosomal lipid metabolism, occurs in glomerular diseases of nongenetic origin (Merscher and Fornoni 2014). However, what factors trigger these lipid accumulations remains unclear and the cause is seldomly traced back to (a) lysosome-dependent pathomechanism(s). Similarly to the proteasomal degradation system, lysosomal proteins and activities are induced in glomerular injury in humans and rodents, however, frequently secondary after the induction of the proteasomal system (Beeken et al. 2014), suggesting a potentially compensatory effect for an underlying proteasomal impairment. Of note, not all lipid accumulating glomerulonephritis forms exhibit a regulation of lysosomes. In human pathology, one example is minimal change disease, where no transcriptional regulation of lysosomal membrane proteins or acid hydrolases occurs, contrasting patients with focal segmental glomerulosclerosis or diabetic nephropathy, where these transcripts are enhanced (Beeken et al. 2014). Despite this differential regulation of lysosomal proteins, all three glomerulonephritis forms exhibit abnormal lipid accumulation (Merscher and Fornoni 2014). Similar observations were made in different rat models of glomerular disease, where puromycin aminonucleosid-induced glomerulonephritis does not result in a transcriptional regulation of lysosomal membrane proteins or acid hydrolases in glomeruli, whereas in the passive Heymann nephritis model of membranous nephropathy such a regulation occurs. Therefore, lysosomal induction does not appear to be a universal reaction to glomerular injury and the factors resulting in / or preventing an induction of lysosomal biosynthesis need to be unraveled for the specific disease entities. Not surprising, a functioning lysosomal system is required for the attenuation of glomerular injury, as shown in the exacerbation of glomerular injury following the induction of antibody-mediated podocyte injury in Limp2-deficient mice (Beeken et al. 2014).

## Specific glomerular diseases

A direct link to lysosomal impairment and glomerular cell injury has been reported in patients with primary podocyte diseases such as membranous nephropathy and focal segmental glomerulosclerosis and in secondary glomerular injuries in the setting of diabetes and ANCA-vasculitis. These will be highlighted in a disease-specific manner in the following sections and in Fig. 4c–f. Other glomerular injuries such as IgA nephritis, membranoproliferative GN are likely to exhibit alterations in lysosomal function; however, no data to such observations are published to the best of our knowledge.

### Membranous nephropathy (Fig. 4c)

Membranous nephropathy is an autoimmune-mediated form of podocyte injury, where autoantibodies bind to the podocyte foot process expressed antigens PLA_2_R1 (Beck et al. 2009) and THSD7A (Tomas et al. 2014) and induce injury (Meyer-Schwesinger, C., Huber, T.B. 2020; Tomas et al. 2016) culminating in nephrotic syndrome. Morphologic hallmarks of MN are a thickening of the glomerular basement membrane, the subepithelial deposition of immune deposits and complement, and foot process effacement. The mechanisms that initiate podocyte injury after autoantibody binding to foot process antigens remain obscure. Complement deposition (Beck 2017) and mechanical or antigen-related effects are discussed (Herwig et al. 2019). Downstream of these initiating events, catabolic pathways are induced in human and rodent MN (Beeken et al. 2014; Meyer-Schwesinger et al. 2009). An impairment of the proteasomal system is thought to relate to podocyte hypertrophy (Lohmann et al. 2014) and injury in MN (Meyer-Schwesinger et al. 2011). An upregulation of lysosomal proteins, especially of Limp2-expressing lysosomes (Rood et al. 2015), and cathepsin D (Wu et al. 2011) is prominent in MN. In light of the fact that signs of lysosomal impairment such as accumulation of lipids and of undegraded storage material in lysosomes are not described in human MN, lysosomal induction might represent a compensatory mechanism for proteasomal impairment (Beeken et al. 2014). Further, lysosomal acid hydrolases were suggested to be involved in the slow degradation of the deposited IgG in MN (Singh 1992). Interestingly, a recent publication by Liu et al. suggests a role for lysosomal dysfunction as the basis of impaired autophagy in MN (Liu et al. 2017). In this study, lysosomal membrane permeabilization (LMP) was detected in cultured human podocytes exposed to sublytic complement C5b-9 membrane attack complex (Liu et al. 2017). Since oxidative stress is prominent in MN (Exner et al. 1996; Neale et al. 1994), the LMP observed in cultured podocytes exposed to sublytic C5b-9 could be the sequel of reactive oxygen species mediating lysosomal injury. It will be of interest to see, if LMP occurs in human MN and whether it accounts for podocyte injury.

### Diabetic nephropathy (Fig. 4d)

Another glomerular injury form, in which oxidative stress is prominent and could induce LMP in glomerular cells is diabetic nephropathy (DN), the most common cause of end-stage renal disease. Glomerular changes in DN include thickening of the glomerular basement membrane, mesangial expansion, proteinuria and fibrosis. All three glomerular cell types are affected in DN, and podocyte damage and loss contribute to the impairment of renal function. Studies showing the importance of mTOR signaling (and thereby autophagy and lysosomal function) in DN are numerous and reviewed in (Li et al. 2020b). Lysosomal protein transcripts are upregulated in glomeruli of patients with DN (Beeken et al. 2014). Lysosomes exert a protective role in DN due to their end-position within the endocytic pathway responsible for the degradation of cargo delivered by autophagosomes. The injury of glomerular cell types in DN is driven by advanced glycation end products (AGEs), which increase reactive oxygen species (ROS) in podocytes (Zhou et al. 2012). On the one side, ROS represent one of the factors inducing autophagy in DN (Ma et al. 2013), which is considered cytoprotective. On the other side, podocytes exposed to AGEs show enhanced oxidative stress, which then triggers lysosomal membrane permeabilization and hence, secondary impairment of autophagy (Liu et al. 2019). The finding that cathepsin L knockout mice are protected from glomerular damage in diabetes (Garsen et al. 2016) supports the concept of LMP as a main trigger to glomerular cell injury in DN. Cathepsin L is one of the acid hydrolases leaking from injured lysosomes in LMP and promotes the proteolysis of proteins essential for foot process morphology, namely CD2AP, synaptopodin (Yaddanapudi et al. 2011), and dynamin (Sever et al. 2007). Besides LMP as a mechanism by which lysosomes drive glomerular cell injury in DN, a decreased interaction of lysosomes with MVBs was suggested to induce lysosome exocytosis of extracellular vesicles containing pro-inflammatory IL-1β (Hong et al. 2019), thereby triggering an inflammatory response. Further, altered lysosome-dependent lipid metabolism contributes to glomerular cell injury in DN. In diabetic mice, lysosomal acid ceramidase, which converts ceramide to sphingosine, is reduced (Choi et al. 2018) and pharmacologic enhancement of lysosomal acid ceramidase activity ameliorates glomerular endothelial cell and podocyte injury (Choi et al. 2018). This could represent a relevant mechanism of human glomerular cell injury, as DN patients exhibit enhanced urinary ceramide content (Morita et al. 2020). Of note: Other metabolic conditions are associated with increased ceramide levels due to decreased lysosomal acid ceramidase levels or activity, such as obesity and hyperhomocysteinemic nephropathy, directly linking metabolism to altered lysosomal function and glomerular cell injury (Li et al. 2020b).

### Crescentic glomerulonephritis (Fig. 4e)

Lysosomes have reached fame in nephrology with the discovery that antibodies to a bacterial antigen can cross-react to a previously characterized antineutrophil cytoplasmic antigen (ANCA), namely LAMP2 (Kain et al. 1995) to cause pauci-immune necrotizing and crescentic glomerulonephritis (Kain et al. 2008). Mechanistically, these autoantibodies activate neutrophils and damage human microvascular endothelium in vitro and cause pauci-immune focal necrotizing glomerulonephritis in rats. This pathogenicity of LAMP2 autoantibodies is thought to arise (1) from autoantibody binding to LAMP2 at the plasma membrane and at the limiting membrane of myeloperoxidase (MPO) and proteinase 3 (PR3) containing lysosomes in neutrophils (Kettritz 2012) and (2) from binding to a structurally related membrane protein at the surface of glomerular and renal microvascular endothelial cells (Kain et al. 2008, 1995). LAMP2, as other lysosomal membrane proteins, is found at low levels at the plasma membrane of cells, a fact most likely attributed to a MPR-independent targeting pathway of lysosomal membrane proteins to lysosomes (Carlsson et al. 1988). The involvement of anti-LAMP2 autoantibodies remains controversial in crescentic glomerulonephritis because of the absence of confirmatory data from other groups regarding their high prevalence and their pathogenicity (Kettritz 2012; Roth et al. 2012).

Besides the abovementioned potential involvement of autoantibodies to LAMP2 in the pathogenesis of crescentic GN, a study performed in Limp2-deficient mice highlights a pro-inflammatory involvement of lysosomes in the anti-glomerular basement membrane model of crescentic GN, as Limp2-deficient mice exhibit a decreased renal infiltration of macrophages and T cells (Lee et al. 2014). This could relate to a blockage of the lysosomal involvement in driving inflammation through lysosomal exocytosis and inflammasome activation.

### Focal segmental glomerulosclerosis (Fig. 4f)

Focal segmental glomerulosclerosis (FSGS) is a generic term for a histological injury pattern defined by segmental glomerular consolidation into a scar that affects some but not all glomeruli with a wide range of etiological interpretations. FSGS describes both a disease characterized by primary podocyte injury (primary FSGS) and a lesion that occurs secondarily in any type of chronic kidney disease (secondary FSGS) (Fogo 2015). Besides the direct involvement of LIMP2 mutations to genetic FSGS, the studies underlining lysosomal involvement in secondary FSGS are rare. An upregulation of lysosomal protein transcripts is seen in glomeruli of patients with FSGS (Beeken et al. 2014); however, the prominent upregulation of LIMP2 observed in MN patients is absent in FSGS (Rood et al. 2015). Nonetheless, the connection between metabolism–lysosomal dysfunction–glomerular injury discussed in the setting of DN is most likely to also contribute to the development of secondary FSGS. Studies of the increased susceptibility of African Americans to FSGS have implicated variants of apolipoprotein L1 (APOL1) (Genovese et al. 2013) to lysosomal dysfunction in podocytes. As a secreted protein, APOL1 can induce lysosomal membrane depolarization and a continuous chloride influx, representing the mechanism by which APOL1 promotes lysis of trypanosomes and imparts resistance to trypanosomiasis (Perez-Morga et al. 2005). Experimental expression of the APOL1 risk alleles in a podocyte-specific manner is causal for podocyte foot process effacement, proteinuria, and glomerulosclerosis (Beckerman et al. 2017). The risk-variant APOL1 alleles interfere with endosomal trafficking and block autophagic flux, ultimately leading to inflammatory-mediated podocyte death and glomerular scarring (Beckerman et al. 2017). Pointing to a mechanistic involvement of lysosomes in this setting is the fact that inhibition of chloride channels and of the interaction of APOL1 with lysosomes attenuates lysosomal swelling and APOL1 variant-induced podocyte injury (Lan et al. 2014).

## Closing remarks

Lysosomes represent the endpoint of numerous vesicle trafficking pathways including the endocytic, phagocytic, and autophagic pathways and physiologically and pathophysiologically represent a cellular node for catabolism and signaling. The lessons we learn from existing investigations on the effect of lysosomal storage disorders and of nongenetic lysosomal disorders on glomerular cell types leave many questions open and stress the urgent need for future basic research. Glomerular cell-type-specific research should focus on (1) lysosomal enzyme targeting mechanisms (endocytosis) to lysosomes; on (2) the distribution of lysosome types; on (3) the dependence on protein and lipid metabolism; on (4) the susceptibility to accumulating macromolecules; and on (5) the stress response mechanisms activated in specific subsets of lysosomal disorders. These investigations are extremely challenging due to the rare nature of LSDs, the difficulty to phenocopy human lysosomal disorders in rodents, the lack of experimental techniques that go beyond the localization and morphology of lysosomes and allow an assessment of lysosomal functionality in archived tissue, and the fact that glomerular cell types and cellular degradation systems are most likely to interact in their quest to keep glomeruli free from macromolecule accumulations. Human-relevant research should be the focus of future investigations, as the profound differences in the cellular metabolism of lipids and proteins between rodents and humans renders knowledge transfer from bench-to-bed challenging. A deeper knowledge in these fundamental processes will pave the way to a better understanding of the clinical heterogeneity lysosomal dysfunction exerts in glomerular cell-types, a prerequisite for the development of specific and efficient therapeutic targeting strategies. It is to be expected that new therapeutic strategies beyond mTOR inhibitors are forthcoming, which specifically target different components of lysosomal function such as the activity of specific lysosomal acid hydrolases, lysosomal Ca^2+^ channels, or which stabilize lysosomes to prevent lysosomal membrane permeabilization.
